# Contactless graphene conductivity mapping on a wide range of substrates with terahertz time-domain reflection spectroscopy

**DOI:** 10.1038/s41598-017-09809-7

**Published:** 2017-09-06

**Authors:** Hungyen Lin, Philipp Braeuninger-Weimer, Varun S. Kamboj, David S. Jessop, Riccardo Degl’Innocenti, Harvey E. Beere, David A. Ritchie, J. Axel Zeitler, Stephan Hofmann

**Affiliations:** 1 Department of Engineering, Lancaster University, Lancaster, LA1 4YW United Kingdom; 2 Department of Engineering, University of Cambridge, J. J. Thomson Avenue, Cambridge, CB3 0FA United Kingdom; 3 Cavendish Laboratory, University of Cambridge, J. J. Thomson Avenue, Cambridge, CB3 0HE United Kingdom; 4 Department of Chemical Engineering and Biotechnology, University of Cambridge, Cambridge, CB2 3RA United Kingdom

## Abstract

We demonstrate how terahertz time-domain spectroscopy (THz-TDS) operating in reflection geometry can be used for quantitative conductivity mapping of large area chemical vapour deposited graphene films on sapphire, silicon dioxide/silicon and germanium. We validate the technique against measurements performed with previously established conventional transmission based THz-TDS and are able to resolve conductivity changes in response to induced back-gate voltages. Compared to the transmission geometry, measurement in reflection mode requires careful alignment and complex analysis, but circumvents the need of a terahertz transparent substrate, potentially enabling fast, contactless, in-line characterisation of graphene films on non-insulating substrates such as germanium.

## Introduction

The development of scalable integrated manufacturing pathways for graphene is crucial to all its emerging applications and industrial development^1^. Chemical vapour deposition (CVD) has become the dominating technique to synthesise large area “electronic-quality” graphene^2^, with the size of single mono-layer graphene crystals now on the cm-scale^3^ and continuous films now routinely produced roll-to-roll or at a size just limited by the reactor^4^. In fact, progress in growth reached a level where detailed, adequate characterisation over such large areas has become a key challenge. Prevailing electrical characterisation for instance is based on the fabrication of field-effect or Hall bar devices typically combined with Raman spectroscopy. This is time-consuming for large samples and for statistically relevant sample numbers in particular also as the as-grown graphene typically has to be transferred away from the growth substrate. Among a range of emerging contactless characterisation methods, terahertz time-domain spectroscopy (THz-TDS) operating in transmission geometry has been demonstrated to allow the direct, accurate mapping of graphene conductivity and mobility over large areas, producing data consistent with the Drude model to describe graphene intra-band transitions^5–10^. Graphene’s complex conductivity is determined by the terahertz pulse transmitted through the graphene film relative to the support, and analysed using Fresnel coefficients where graphene is modelled as an infinitely thin conducting film. Drift and field-effect mobilities can then be extracted by fitting the conductivity spectra to the Drude model^9^, and measuring graphene conductivity changes as a function of the applied back-gate voltages on a gate-stack support^8^, respectively. By repeating the measurement and analysis across the entire graphene area, a conductivity or mobility map can be reliably produced. While these demonstrations could potentially enable a rapid in-line graphene monitoring and large-area characterisation, to date THz-TDS has been carried out in transmission mode that necessitates a terahertz transparent support.

**Figure 1:**
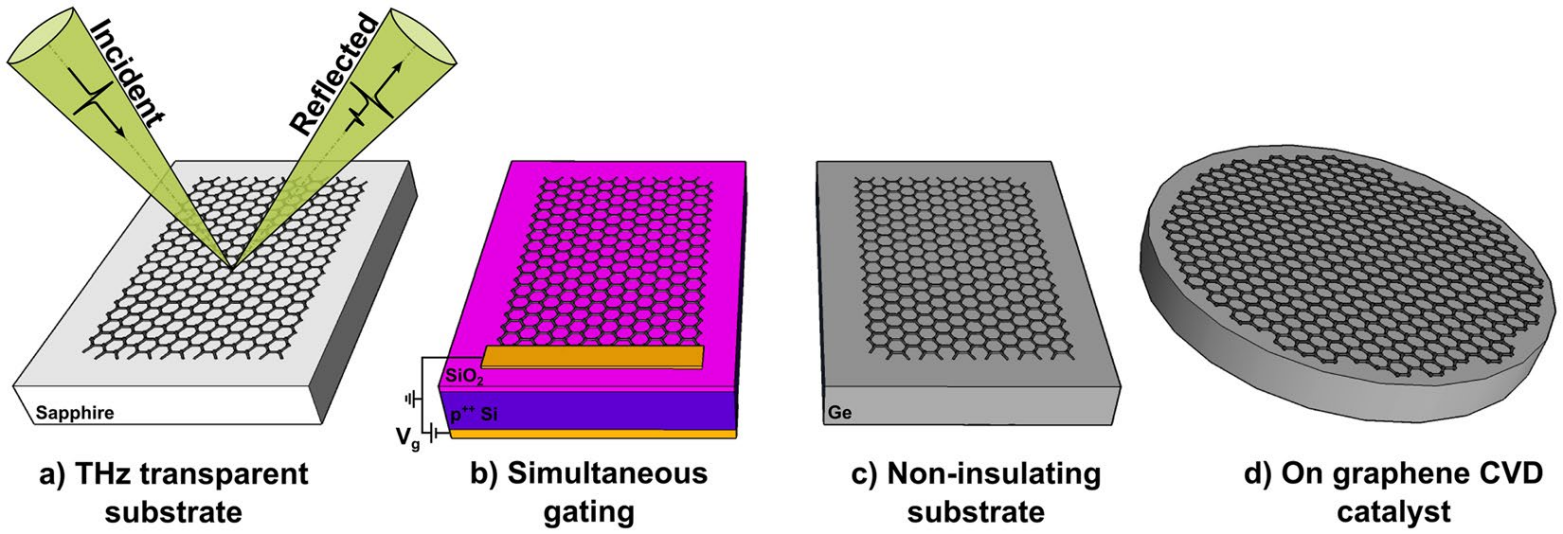
Schematic of THz-TDS setup where pulsed terahertz radiation is directed at graphene on a range of substrates: (**a**) sapphire support to allow terahertz reflection and transmission measurements used for the initial validation of our method, (**b**) boron-doped Si/SiO_2_ device substrate with simultaneous back-gating, (**c**) intrinsic Ge support and (**d**) Ge(110) growth substrate to demonstrate in-line characterisation without graphene transfer.

Here we overcome this restriction and demonstrate, as a proof-of-concept, the quantitative contactless measurement of the electrical conductivity of CVD graphene on a range of application relevant supports using THz-TDS operating in reflection geometry. Reflection based THz-TDS has previously been used to characterise optically dense materials where due to the high energy loss within the sample, transmission based measurement cannot be used^11–15^. As schematically outlined in Fig. 1, we first validate our measurements in reflection geometry by performing the more well-established terahertz transmission conductivity mapping^5, 7, 10^ on the same graphene sample. For this we use CVD grown graphene synthesised on commercial Cu foils and transferred to a sapphire substrate, which has the required transparency in the THz frequencies. We then show that by sample back-gating, our proposed method can be used to resolve graphene conductivity changes and hence to directly determine the graphene mobility on a p-doped Si substrate with a 300 nm thick SiO_2_ layer, which is one of the most commonly used substrates for graphene device manufacturing. To illustrate that this technique has potential as a tool for in-line graphene quality monitoring, we show that the graphene conductivity can also be directly mapped on a substrate like Ge, which has a considerable number of intrinsic carriers at room temperature (42 Ω·cm) and exhibits a Drude like carrier absorption^16^. In particular we demonstrate THz-TDS mapping for graphene that has been directly grown on Ge.

## Methods

### Graphene growth and transfer

As reference process and material, we use well-established graphene CVD on commercial Cu foil and subsequent PMMA transfer, which is widely used in literature^17^. We use standard Cu foils (25 μm thick, Alfa Aesar purity 99.8%) and CH_4_ as the carbon precursor^18^. For transfer, PMMA (poly methyl-methacrylate) was used as support, followed by FeCl_3_ chemical etching to remove the Cu. As target substrates we used sapphire (430 μm thick), Si/SiO_2_ wafer (300 nm/525 ± 25 μm thick), and intrinsic single crystalline Ge (110) wafer (50 Ω·cm, from MTI Corporation). Raman spectroscopy was performed using a 532 nm laser for characterising the transferred graphene. The Si wafer support was boron-doped (100 Ω·cm) to allow back-gating. The process conditions for graphene CVD directly on Ge were similar to recent reports in literature^19^ and we used Ge (110) wafer substrates and a 1:52 precursor gas mixture of CH_4_/H_2_ at a growth temperature of 920 °C in a Aixtron Black Magic cold wall CVD reactor.

### Terahertz time-domain reflection spectroscopy

Reflection based THz-TDS experiments were conducted with a Terahertz Pulsed Imaging (TPI) Imaga 2000 system (TeraView, Cambridge, UK), as schematically shown in Fig. 1. The terahertz radiation used here is broadband, covering a spectral range of 0.15–3 THz in free-space. Terahertz radiation is generated by pumping a biased photoconductive antenna with an ultrashort laser pulse from a Ti:Sapphire laser. The emitted terahertz pulse is collected, collimated, and then focused onto the sample with a focal length of 7 mm at an incident angle of 30°. The reflected terahertz pulse is then collected and focused onto an unbiased photoconductive antenna for the laser-gated terahertz detection. The TPI achieves a spatial resolution of approximately 400 μm at 1 THz^20^ that in turn allows us to estimate the spot size of approximately 420 μm with Gaussian beam optics^21^. One of the main barriers for accurately extracting optical parameters in reflection geometry is the great sensitivity to any phase misalignment between the sample and reference measurements. In general, the phase misalignment can be mitigated by performing additional measurements with a slab of material of known optical constants^14^, maintaining same path length change for both terahertz and optical beam^15^ and numerical correct phase correction using the maximum entropy method^22^. Attention was given to precise sample positioning by having both the Al mirror and the sample mounted on a motorised stage and positioning the respective front surfaces in order to ensure that the particular reflecting plane reflects the incident wave into the detector at maximum level^12^. It should also be noted that the substrate refractive index measured is not adversely affected by phase misalignment, as in the case for the extinction coefficient^13^. As an experimental check, the measured substrate refractive index for s-polarisation^23^ was always compared against the literature values. The reflection coefficient depends on the polarisation of the incident terahertz wave and the angle of incidence. Given the substrate refractive index and using Tinkham’s formulae to describe the effect of a thin conducting film^24^, the equation for obtaining conductivity from s-polarisation reflection measurements can be derived as:

1 $$\tilde{r}=\frac{{\tilde{n}}_{1}cos{\theta }_{i}-{\tilde{n}}_{2}cos{\theta }_{t}-{z}_{0}\tilde{\sigma }}{{\tilde{n}}_{1}cos{\theta }_{i}+{\tilde{n}}_{2}cos{\theta }_{t}+{z}_{0}\tilde{\sigma }}$$

where $\tilde{r}$ is the Fourier transformed ratio of the reflected wave’s complex electric field from the sample to the incident wave from the mirror, z_0_ is the vacuum impedance (376.7 Ω), *θ*_*i*_ and *θ*_*t*_ are the incident and transmitted angles, respectively, and ${\tilde{n}}_{1}$ and ${\tilde{n}}_{2}\,\,$are the complex refractive indices of air and substrate, respectively^25, 26^. At normal incidence and assuming zero conductivity, we get the well-known Fresnel coefficient for the reflection at the interface:

2 $$\tilde{r}=\frac{{\tilde{n}}_{1}-{\tilde{n}}_{2}}{{\tilde{n}}_{1}+{\tilde{n}}_{2}}$$

The analysis becomes simpler at normal incidence but such a reflection system in turn would require a beamsplitter that is not commonly implemented due to reflection losses^27^.

Using Snell’s law $cos{\theta }_{t}=\sqrt{1-\frac{si{n}^{2}{\theta }_{i}}{{\tilde{n}}_{2}^{2}}}$, the complex conductivity can be expressed as

3 $$\tilde{\sigma }=\frac{{\tilde{n}}_{1}cos{\theta }_{i}(1-\tilde{r})-(1+\tilde{r})\sqrt{{\tilde{n}}_{2}^{2}-si{n}^{2}{\theta }_{i}}}{{z}_{0}(1+\tilde{r})}$$

We performed analytical simulations to double check the derived expressions and optical constants, whereby the simulations involved (1) simulating a terahertz pulse generated from a photoconductive antenna switch with realistic parameter settings such as 120 fs, 300 fs and 180 fs being the laser pulse duration, emitter/detector carrier recombination and collision time, respectively^28^; (2) determining reflections from graphene and the substrate using the Fresnel coefficients, the real part of the materials refractive index in the literature^16^ and a constant graphene conductivity value for CVD graphene grown on Cu foil^7, 10, 29^; and (3) applying the derived expressions to obtain conductivity for comparison against the conductivity defined in step 2. The use of only the real part of the materials refractive index is justified by the use of substrate materials such as sapphire and intrinsic Ge that have a negligible extinction coefficient in the relevant frequency range between 0.5 to 1 THz^16^. The simulation highlighted that, where there is a phase shift deliberately introduced between the sample reflection and the reference reflection, the slope of the derived conductivity spectra is no longer zero (see Supporting Information). This fact is exploited in the automated analysis of our measurement for phase correction. It should be noted that phase misalignment can be due to several reasons, such as fibre drifts and mechanical jittering of the optical delay stage, and that these problems are most severe in fibre-coupled terahertz systems as used in this study. Our phase correction method therefore shifts the acquired reflection pulse with respect to the reference measurement in the time-domain in order for the real part of the calculated conductivity spectra to have a slope close to zero. A single step shift in time here corresponds to a sample being placed approximately 2.5 μm with respect to the reference mirror, and a positive value means that the sample position is shifted in the direction of the incident beam. We note that the phase compensation scheme can be alternatively implemented in the frequency domain by multiplying the phase shift term^6^.

Transferred graphene on sapphire was measured with TPI at a step size of 200 μm, where 15 waveform traces were averaged to represent a measurement for one single pixel. Here the number of waveforms acquired for averaging is relatively small as would be the requirement for potential in-line applications. Before the measurement, however, terahertz reflection from the Al mirror placed nominally at the same position as the sample was used as reference measurement. An Al mirror generally works as an almost perfect reflector in the terahertz regime^30^. From the raster scanned measurement, the region of the substrate covered by graphene was isolated by masking the data with an intensity threshold value given that the graphene covered area corresponds to regions of higher reflectivity relative to the plain sapphire substrate. As the primary reflection was well separated from the first reflection in the time-domain, a time windowing function was used to process the acquired waveforms in the regions of interest in order to remove Fabry-Perot or etalon effects that would otherwise corrupt the conductivity measurement. A conductivity map was subsequently generated. In order to validate our proposed method, the same sample was scanned with THz-TDS operating in transmission mode. In particular, measurements were acquired with the Tera K15 T-Light (Menlo Systems GmbH, Germany) where the 60 mW pump pulse was focused to a 40 μm spot onto the terahertz photoconductive antenna, generating terahertz radiation with a beam diameter of approximately 1 mm at 1 THz. The sample was placed between the terahertz emitter and the detector at normal incidence without nitrogen purge, and data were acquired at an integration time of 10 ms at 200 μm step intervals. The integration time constant corresponded to an average of 700 waveform traces. The region of the substrate covered by graphene was again obtained by intensity masking, where graphene covered areas correspond to regions with a reduction in transmitted intensity due to the higher carrier absorption in graphene^10^. By performing the analysis detailed in recent literature^7, 10^, a comparative conductivity map for approximately the same sample area was generated.

### Data availability

Additional data sets related to this publication are available from the Cambridge University data repository at https://doi.org/10.17863/CAM.12742.

## Results and Discussion

### Graphene conductivity on sapphire substrate

**Figure 2:**
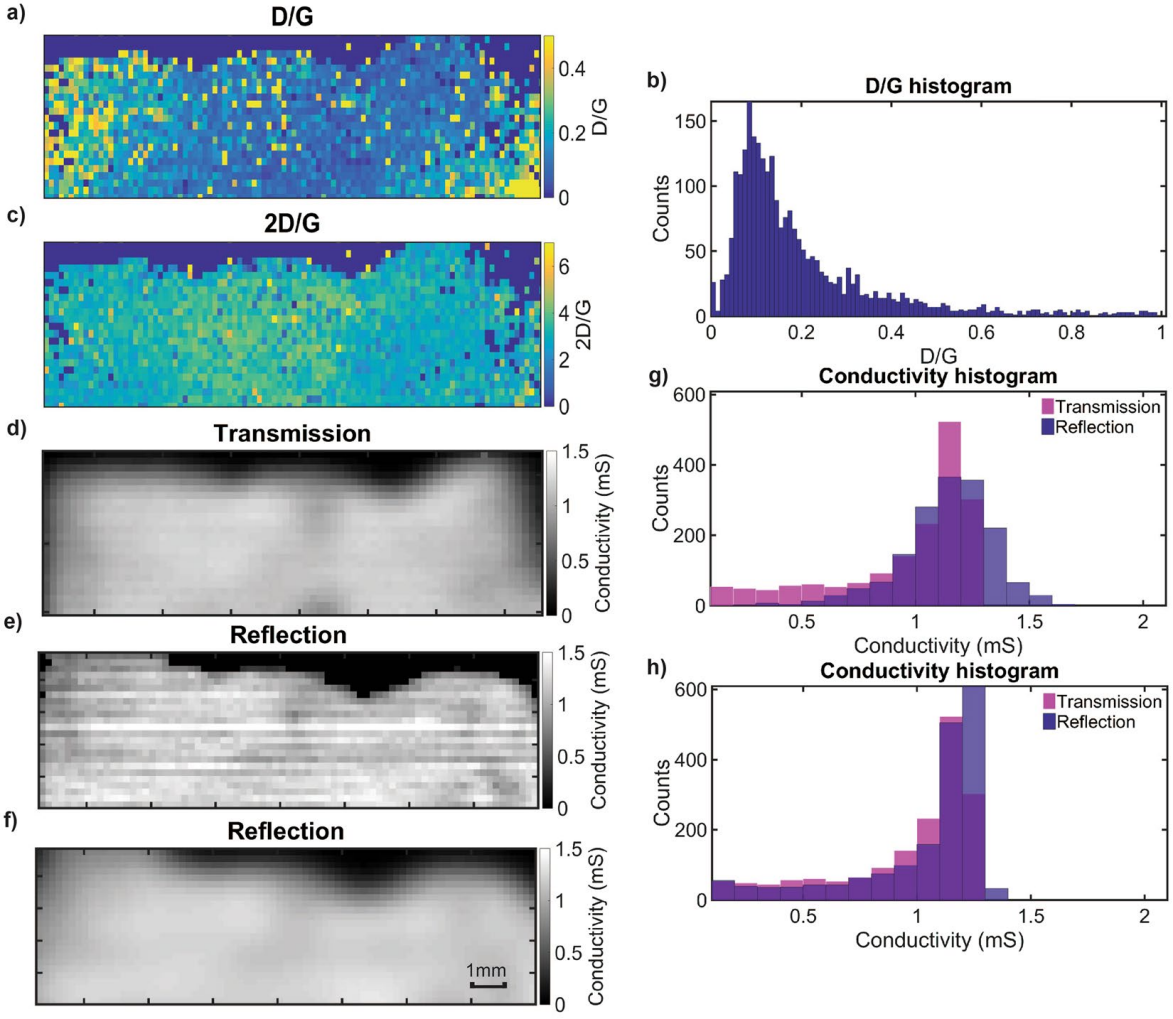
Raman map of graphene on sapphire substrate, (**a**) D/G ratio map, (**b**) D/G frequency distribution, (**c**) Raman 2D/G map. For the same region a conductivity map of graphene on sapphire substrate was measured with THz-TDS between 0.6–0.9 THz operating in (**d**) transmission mode with a Tera K15 T-Light setup and (**e**) in reflection mode with TPI, where (**f**) shows a spatially filtered map of (**e**) with a spot size 2.4 times greater. Conductivity histograms for transmission and reflection geometries are compared in (**g**) before and (**h**) after filtering. The colour of the histogram is darkened at the overlap between reflection and transmission measurements. Raman and terahertz mapping both resolve a similar shape of the transferred graphene film.

Figure 2a–c shows the Raman graphene D/G ratio map, frequency distribution, and 2D/G ratio map of the transferred graphene on sapphire. Most measured points show a D/G ratio of roughly 10%, highlighting the presence of defects that have been introduced during the transfer procedure. The 2D/G ratio map shows an average value of more than 1 highlighting that the film is predominately monolayer graphene. The substrate refractive index measured in transmission mode TDS is 3.1, in close agreement with literature^16^. This value was then used to obtain flat conductivity spectra^7, 10, 29^ (see Supporting Information) without any phase correction, which in turn was used to generate the transmission conductivity map shown in Fig. 2d. The imaginary conductivity measured here is no longer negligible due to the fact that phase was not accounted for. Graphene film conductivity measurements with THz-TDS in transmission geometry have previously been benchmarked against micro-four point probe, micro Raman spectroscopy and optical imaging^7^. Here we use transmission measurements to validate our measurements in reflection geometry. The substrate refractive index measured in reflection mode was approximately 3. The slight discrepancy may be due to sample surface related imperfections, leading to scattering losses and measurement with a focused beam as opposed to a collimated beam^31^. In a manner similar to the literature^7, 10, 29^ and our transmission measurements, the graphene conductivity spectra on sapphire for a well-aligned reflection measurement have a real part characterised by a flat spectral response near to its DC value well below the Drude roll-off frequency or the inverse scattering time, while the imaginary part is close to zero (see Supporting Information). The spectrally resolved conductivity in turn can be represented by a single real number. It has been shown that phase misalignment would only significantly affect the imaginary part of the conductivity^6^ and therefore emphasis is placed only on the real part of the conductivity. As the conductivity spectra are approximately constant over the spectral range, the representative conductivity is taken as the average between 0.6 to 0.9 THz because at higher frequencies, the transmission conductivity spectra (see Supporting Information) become affected by water vapour absorption under ambient conditions. This effect become visibly more pronounced with increasing optical path length. Similarly, for the conductivity measurement in reflection mode, the representative pixel conductivity is taken as the average over the same spectral range of 0.6 to 0.9 THz.

Figure 2 compares the conductivity map and histogram obtained with terahertz transmission and reflection mode TDS, respectively. The conductivity map acquired in reflection mode contains intermittent interlacing artefacts between alternate rows on the image. This is due to the small signal fluctuations that propagate to the conductivity calculation. Sources of signal fluctuations include fibre drifts, laser instability, optical and electronic noise. Nevertheless, qualitatively, an agreement between the two measurement geometries can be seen, for instance by looking at the regions of low local conductivity. When comparing the histogram in Fig. 2g, the conductivity frequency distribution is generally in agreement despite differences in the spot sizes of the THz-TDS systems used. In order to allow for a comparison that accounts for the differences in terahertz spot size, spatial filtering was applied to the reflection conductivity map to emulate a spot size approximately 2.4 times greater in order to generate a spatially averaged conductivity map and histogram shown in Fig. 2f and h, respectively. This results in a much closer agreement against the transmission measurements. The low conductivity values in the histogram correspond to the pixels positioned close to the graphene boundary and the slightly higher conductivity in reflection compared to transmission may be due to the averaging of the aforementioned signal fluctuations. The demonstration of the reflection measurement here means that, in principle, conductivity can be reasonably estimated directly from the first transmitted pulse, recently demonstrated^6^, as opposed to the first echo in a transmission measurement, even though the measurement robustness is lower for direct transmission analysis^7, 10^. Overall, we were able to validate our proposed approach with good agreement against established transmission measurements.

### Graphene mobility

**Figure 3:**
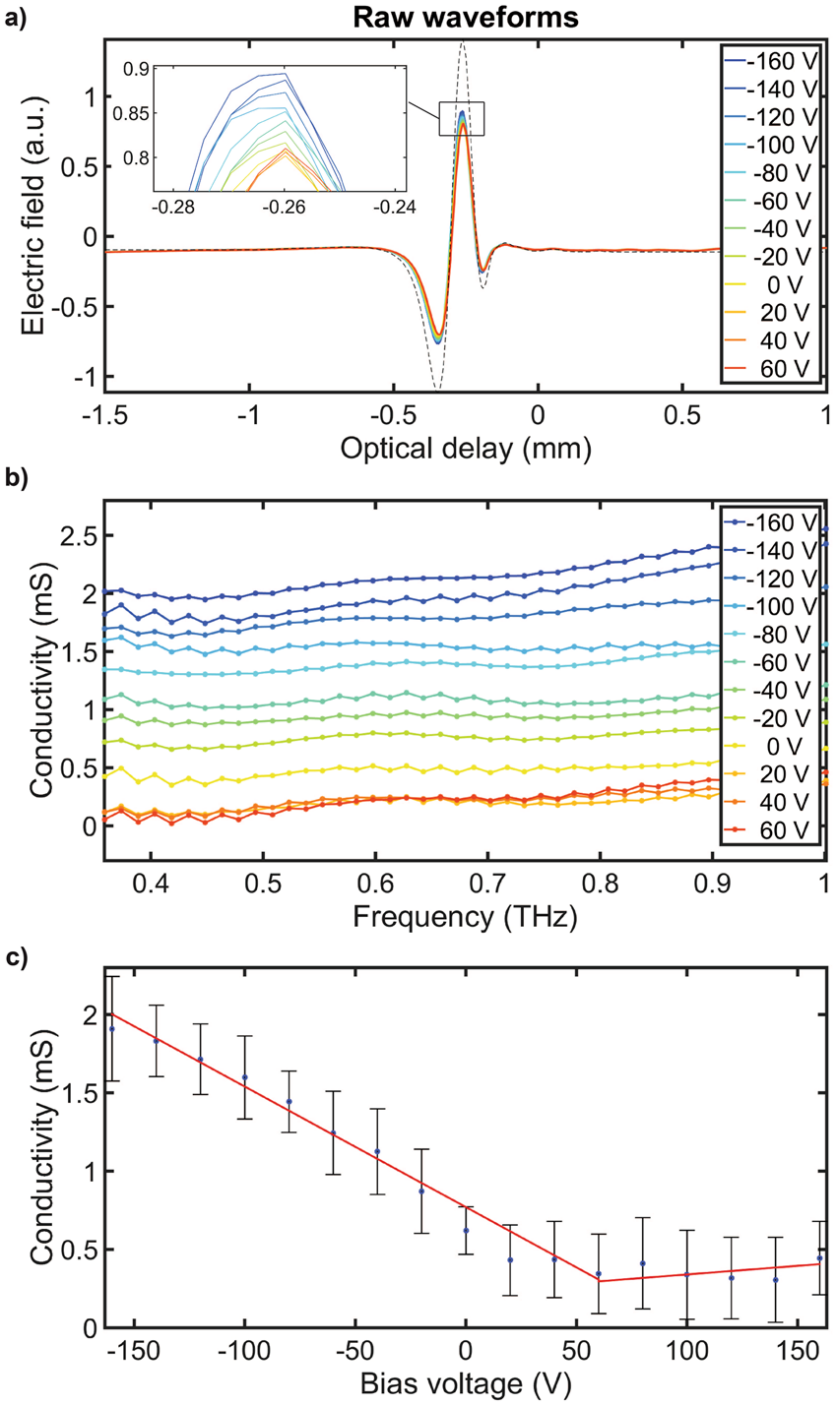
The measured terahertz reflection from a randomly selected position on the graphene on boron-doped Si/SiO_2_ substrate as function of applied back-gate voltage in (**a**) and an expanded view on the peak of the terahertz reflection in (**b**). The corresponding real conductivity spectra in (**c**) and the average real, gate-induced conductivity from 0.4 to 0.9 THz as a function of V_g_ for 3 distinct positions on the graphene area. Circles are experimental data and the lines are linear fits to the data for −160 V < V_g_ < 60 V and 60 V < V_g_ < 160 V for field-effect hole and electron mobilities, respectively.

In order to demonstrate that our proposed method can be used to resolve graphene conductivity changes, we implemented simultaneous back-gating^19, 32^ via a graphene film transferred to a p-doped Si substrate (Fig. 1, see methods). Unlike previous work where complex transistor and Hall bar devices were realised to measure the mobility^33, 34^, here we attached a piece of Cu foil to the Si side of the graphene/wafer-stack and another Cu foil to the edge of the graphene. This rather simplistic setup defined the electrodes for back-gating and grounding the graphene film, respectively. The Fermi level of graphene was electrically tuned via the back-gate voltage supplied by a DC variable voltage supply (Keithley, Model 2400) where various different DC voltages between −160 to 160 V were applied. The back-gate leakage current was negligible. At a fixed point on graphene and at every 20 V back-gate voltage increments, reflection measurements were acquired on the TPI at 15 waveforms per average. The acquired waveforms were then analysed and the sequence of steps were then repeated for 2 other randomly selected locations on the graphene. The measurements were performed in ambient conditions at room temperature. Figure 3a shows a selection of the reflected waveforms, and the expanded views on the signal near the peaks against the applied back-gate voltage. From Equation 1, it is expected that terahertz reflection increases with increasing graphene conductivity induced by electrical gating. The reflection changes were not due to the influence of the substrate as no changes to the waveforms were observed when the terahertz spot was on the substrate under similar gating voltages. At V_g_ between 40 and 60 V, reflection is the weakest indicating that the Fermi energy at this gate voltage is closest to the Dirac point. At all other voltages below 40 V terahertz reflection increases monotonically with V_g_ as shown in Fig. 3b. Based on the acquired terahertz reflection waveforms, the real part of the frequency dependent conductivity was determined, as shown in Fig. 3b. This shows a strong dependence on the applied gate voltage. It can also be observed that there is a slight increase in the conductivity with increasing frequency. This may be due to a small degree of preferential back-scattering of charge carriers in the graphene^8^. Due to the aforementioned reasons, the spectrally resolved conductivity is represented by a single real-value conductivity value taken as the average conductivity between 0.4–0.9 THz. Figure 3c shows the measured conductivity for 3 randomly selected points on the graphene as a function of the applied back-gate voltage. Conductivity shows a linear dependence on the applied gate voltage in the range of −160 ≤ V_g_ ≤ 60 V representing the field-effect mobility of the hole-carriers. For V_g_ > 60 V, a slow conductivity change is observed indicating that electron-mobility is impaired in this graphene film. Linear curve-fitting was used to extract the line slope to determine hole-mobility. A R^2^ value of 0.986 was achieved in the fit that corresponded to a conductivity change of 0.0083 mS V_g_^−1^. This value was then used to determine a mobility of 721 cm^2^V^−1^s^−1^, where ${C}_{ox}=\frac{{\varepsilon }_{ox}}{{t}_{ox}}=11.5\times {10}^{-9}\,Fc{m}^{-2}$ for the 300 nm thick SiO_2_ gate oxide. The extracted back-gated mobility value is comparable to the mobility values extracted from graphene transistor measurements of graphene films prepared in a similar manner^33, 34^. Air-exposed graphene on SiO_2_ is typically found to be p-doped^34^, consistent with the charge neutrality point V_CNP_ at a positive bias^34^. Even though not demonstrated here, mobility mapping with terahertz reflection measurement across the sample could in principle be performed by raster scanning the sample at different back-gate voltages.

### Graphene conductivity mapping on Ge

**Figure 4:**
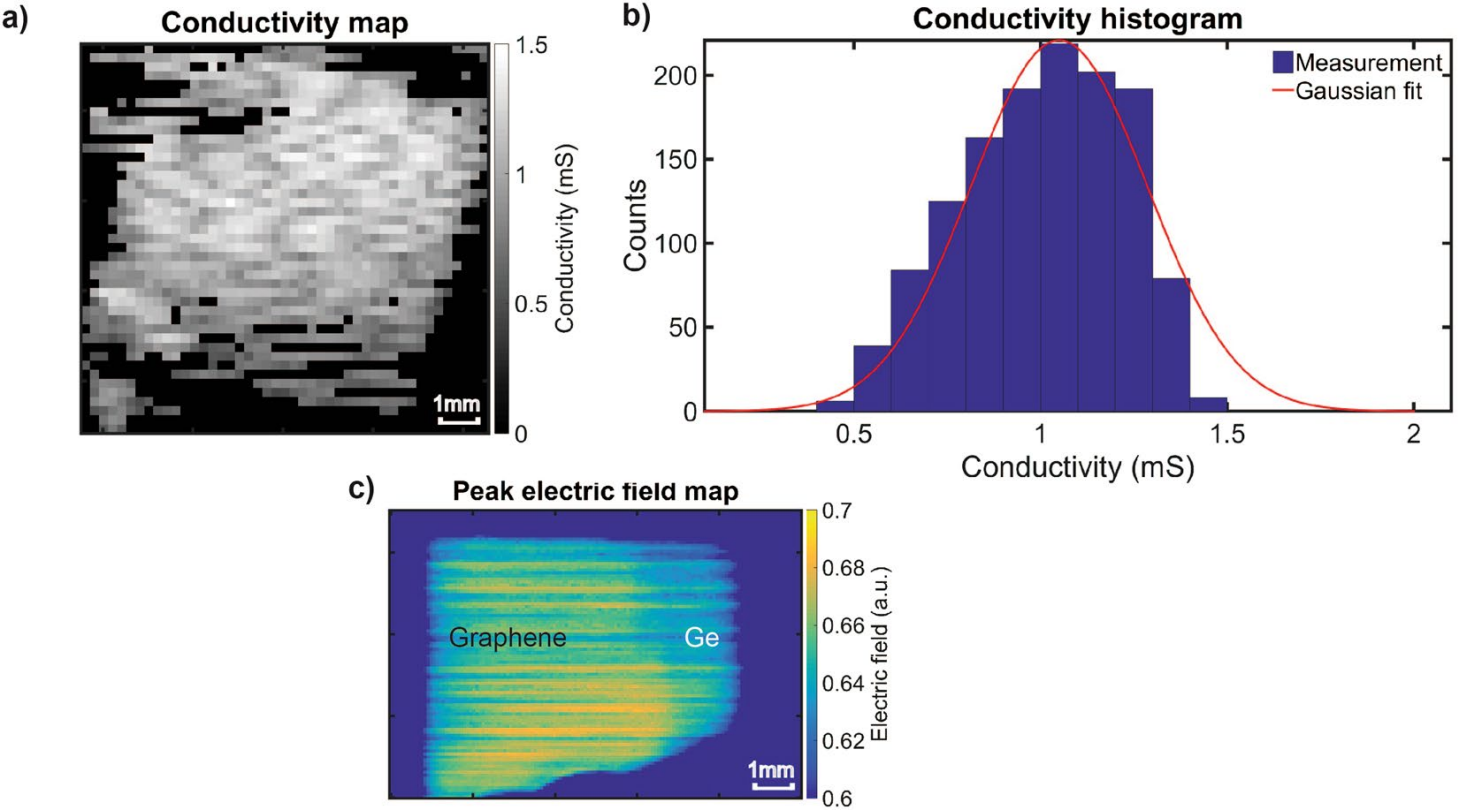
Conductivity map (**a**) of graphene film transferred on Ge(110) substrate measured with TPI and (**b**) the corresponding histogram. (**c**) The peak terahertz electric field map of patterned CVD graphene synthesised directly on Ge(110).

Utilising a reflection rather than a transmission geometry allows contactless mapping of the graphene conductivity on a wider range of substrates. In terms of integrated graphene CVD manufacturing and process optimisation, a clear need is to probe graphene at each process step. We here so far discussed two examples of graphene conductivity measurements after graphene transfer on substrates like sapphire and Si/SiO_2_, but other substrates such as flexible polymers are in principle possible too provided sufficient contrast can be observed, which is generally the case as the real part of the refractive index is less than 2 and the extinction coefficient is negligible at less than 2 THz^35, 36^. This has been highlighted already for transmission measurements^7^. The challenge of adequate characterisation of the graphene still on the growth catalyst/support however remains. For instance, even individual Raman measurements of graphene on transition metals can be challenging, not to speak of mapping large graphene areas. As a first step towards direct contactless graphene conductivity mapping on technologically relevant growth substrates, we here focus on Ge substrates. As a starting point we first transferred graphene on intrinsic Ge(110), and then applied our method to graphene directly grown on Ge(110). The Ge (110) orientation is chosen because it yields higher quality graphene under the CVD process used here^19, 37^. For our initial analysis, the imaginary part of the complex refractive index of Ge can still be assumed to be negligible. For the undoped Ge(110) reference substrate, the graphene conductivity can be expected to be close to the values measured on sapphire, except some minor deviations based on, for instance, charge transfer between graphene and Ge^38^. Hence we can also assume that the real conductivity spectra remain constant over the THz spectral range^7, 10, 29^. For reflection measurements on highly doped Ge, the assumption of a negligible extinction coefficient will no longer hold^39^ and therefore the frequency dependent extinction coefficient needs to be accounted for in the conductivity analysis. Accurate measurement of the extinction coefficient in turn would require very high precision alignment between the sample and reference^13^. Analogous to our measurements of graphene on sapphire, the graphene film transferred on Ge was measured with TPI at a step size of 200 μm where 15 waveform traces were averaged. A conductivity map was then generated from the raster scanned measurement by applying the aforementioned analysis where the region of graphene coverage was obtained by intensity masking the data, where the graphene covered area corresponds to regions with higher reflection relative to Ge. The measured substrate refractive index of Ge was approximately 4 in close agreement with literature^16^. It should be noted that the contrast i.e. reflection change from bare Ge substrate to graphene covered area is approximately 5% for graphene with conductivity of 1.2 mS. This can be compared against graphene on sapphire where for the same conductivity, the contrast is approximately 10%, allowing a clear discrimination between graphene covered areas and the bare substrate. With a lower contrast, the measurement becomes more susceptible to noise and therefore we defined the threshold for intensity masking by considering an average of the substrate reflection. Figure 4a shows the measured conductivity map of transferred graphene on Ge(110) (see Supporting Information for corresponding optical microscope image). Here the blacked out pixels in the region of interest correspond to pixels where the conductivity was not computed because the reflection was below the defined threshold. For all other pixels, the average conductivity over 0.6 to 0.9 THz was estimated (see Supporting Information). On the conductivity map, there are visible spots of low conductivity in part due to the small signal fluctuations as a result of the lower contrast or from the graphene film, though the former is more likely. The conductivity histogram in Fig. 4b shows that the distribution does not closely follow a Gaussian function, but is centred around 1 mS in agreement with the conductivity value measured on sapphire support (Fig. 2). The lower average conductivity may be due to the aforementioned reduced contrast and hence increased sensitivity to the noise inherent to commercial fibre-coupled THz-TDS systems, as well as a smaller graphene covered area leading to the increased influence of boundary areas where lower graphene conductivity is generally observed. There is also qualitative agreement in the shape of the histogram compared to Fig. 2g where a sharp roll-off is observed for high conductivities as opposed to the lower conductivity values. Overall, the Ge measurements show that graphene film conductivity mapping by terahertz reflection spectroscopy on lightly doped substrates is possible, with the obtained conductivity values in agreement to our sapphire measurements. Note that the conductivity value is expected to vary slightly when comparing graphene on sapphire and Ge due to substrate induced doping^38^.

As final validation, we apply our method to graphene directly grown on Ge(110). After CVD on Ge of a uniform graphene coverage (see Methods), we subsequently used drop casted PMMA photoresist and oxygen plasma etching (with oxygen partial pressure 50 mbar at 50 W for 8 s) to define graphene patterns to allow for reference contrast against the plain underlying Ge. After oxygen plasma etching, the remaining photoresist was removed with acetone. We then measured with TPI at a step size of 75 μm where 50 waveform traces were averaged for one pixel. Figure 4c shows the peak electric field map where a contrast of approximately 5% can be observed between the graphene and the exposed underlying Ge. After graphene CVD, the Ge substrate appears to be highly doped (0.25 Ω·cm) as verified by standard conductivity measurements. This therefore means that although qualitative analysis of the intensity map is possible, a quantitative analysis to obtain the conductivity value cannot be reliably performed on this sample because the underlying assumption of negligible extinction coefficient is no longer valid. For the accurate determination of the extinction coefficient, high precision sample alignment would be required for substrate Drude model fitting that is outside the scope of this paper. The proposed technique, however, would be possible for lightly doped substrates (1–10 Ω·cm) where the extinction coefficient still remains negligible for frequencies greater than 0.5 THz as in the case of Si^40^. In principle, these substrates could also work with transmission THz-TDS, though the measurement may slightly overestimate the conductivity due to increased carrier absorption as the terahertz pulse makes a return path inside the substrate. The fact that there is an observable contrast in the reflection measurement is promising for future conductivity mapping of graphene synthesised directly on Ge. It can be expected that in samples where contrast is lower, either because of a higher substrate refractive index at terahertz frequencies, a lower graphene conductivity, or a combination thereof, the conductivity measurement in reflection geometry becomes increasingly susceptible to the effect of signal fluctuations and hence become less reliable. For instance, on growth substrates such as Cu^2^, no contrast could be observed relative to Cu for a sample of similar graphene conductivity because of the high Cu conductivity (59 MS/m) leading to a refractive index (~730) at least two orders of magnitude larger than that observed for sapphire or Ge at 1 THz. At the same time, it should also be pointed out that with the possibility of making changes to the graphene manufacturing process, such as intercalation of a micron-thick oxide layer^41^, these changes potentially could be useful to increase the measurement contrast for future in-line characterisation of graphene on e.g. Cu.

## Conclusion

In summary, we have demonstrated the feasibility and potential of measuring the electrical conductivity of CVD graphene with THz-TDS in reflection geometry. Using terahertz transparent sapphire support, we have validated the technique against current state-of-the-art THz-TDS transmission measurements, where after taking into account the differences in terahertz spot sizes, we find a close agreement between the conductivity histograms. Using back-gated Si/SiO_2_ support, we have further demonstrated the sensitivity of the technique to resolve conductivity changes during electrostatic gating and hence the ability to directly determine graphene mobilities, whereby our values were consistent with standard electrical measurements. To illustrate that this technique has the potential as a tool for in-line graphene quality monitoring, we showed that the graphene conductivity can also be directly mapped on a substrate like Ge, where only half the contrast is seen relative to the sapphire substrate due to an approximately 30% increase in refractive index in the relevant frequency range. For the graphene films synthesised directly on Ge(110), the Ge substrate became highly doped after graphene synthesis and hence high precision alignment is required to accurately determine graphene conductivity. It should be noted that the proposed technique also assumes flat conductivity spectrum, which is not the case for all graphene samples, such as graphene grown on a single Cu crystal. For these samples, provided the shape of the conductivity spectra is known, a similar slope fitting method could be used. A more robust approach, however, would involve high precision alignment that is the subject of ongoing research. Our data shows that while measurement and analysis are understandably more complex and can be less robust when measuring in reflection compared to transmission, especially considering that there is less terahertz interaction with the sample, terahertz time-domain reflection spectroscopy is a highly interesting contactless, quantitative characterisation technique with clear potential to complement existing characterisation techniques for graphene and other related 2D materials and to open new opportunities for the rapid screening of large-area 2D crystals and films, which is crucial towards emerging applications and industrial development of these materials.

## Electronic supplementary material

Supplementary information
