# Supplementary material for: Contactless graphene conductivity mapping on a wide range of substrates with terahertz time-domain reflection spectroscopy
Source: Sci Rep. 2017 Sep 6;7:10625. doi: 10.1038/s41598-017-09809-7 (PMC5587735; doi:10.1038/s41598-017-09809-7)
Supplement: Supplementary file 1 — Supplementary information [file 41598_2017_9809_MOESM1_ESM.doc]

**Supplementary Information for ‘Contactless graphene conductivity mapping on a wide range of substrates with** **terahertz time-domain reflection spectroscopy’**

Hungyen Lin^1*^, Philipp Braeuninger-Weimer^2*^, Varun S. Kamboj^3^, David S. Jessop^3^, Riccardo Degl’Innocenti^3^, Harvey E. Beere^3^, David A. Ritchie^3^, J. Axel Zeitler^4^ and Stephan Hofmann^2^

^1^Department of Engineering, Lancaster University, Lancaster LA1 4YW, UK

^2^Department of Engineering, University of Cambridge, J. J. Thomson Avenue, Cambridge CB3 0FA, UK

^3^Cavendish Laboratory, University of Cambridge, J. J. Thomson Avenue, Cambridge CB3 0HE, UK

^4^Department of Chemical Engineering and Biotechnology, University of Cambridge, Cambridge CB2 3RA, UK


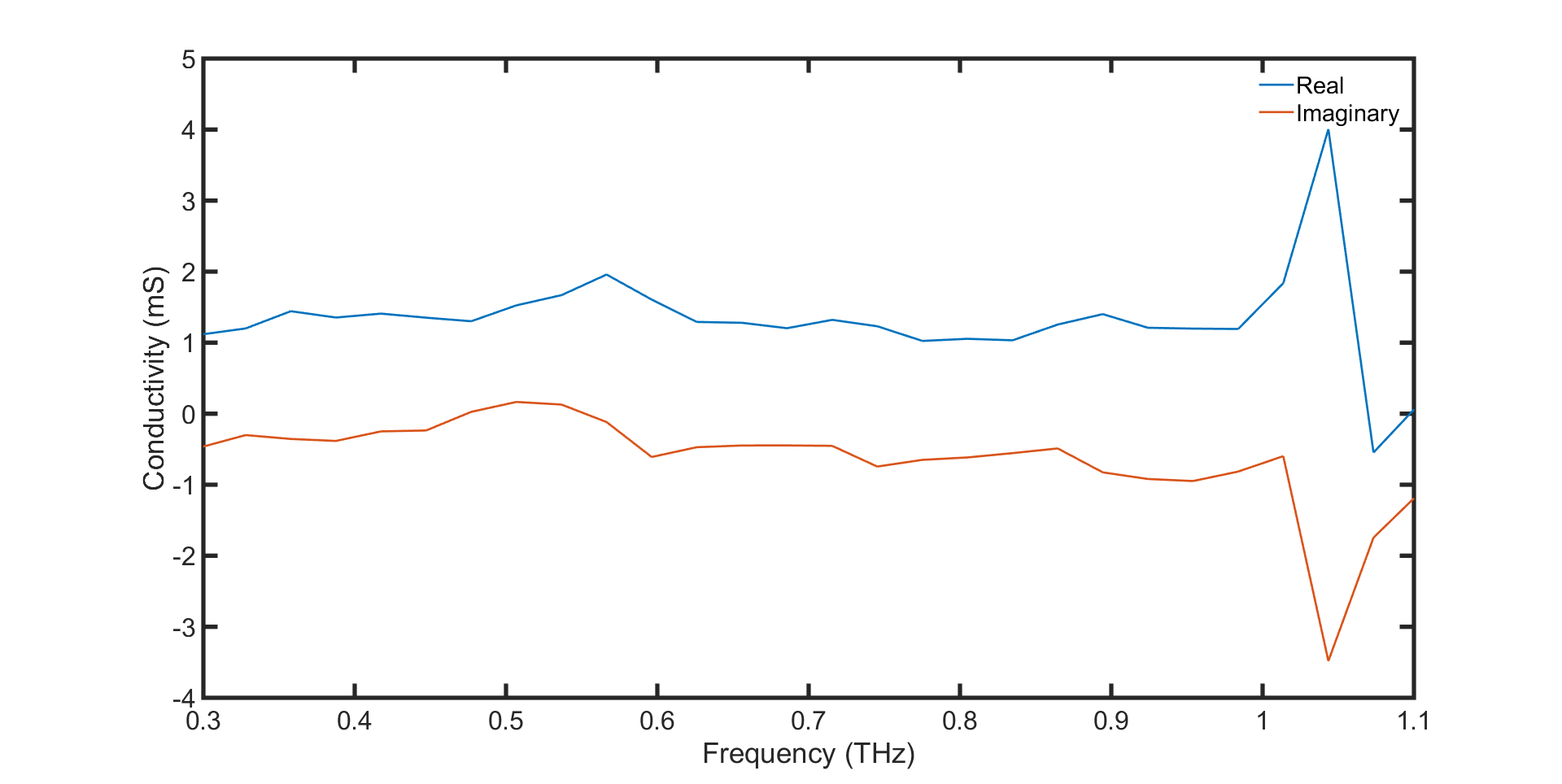


Figure S1 – Conductivity spectra measured with THz-TDS operating in transmission-mode without phase correction, where the increase in conductivity at 0.56 and 1.05 THz is due to water vapour absorption in the atmosphere under ambient conditions. Spectral range is therefore taken as 0.6-0.9 THz.
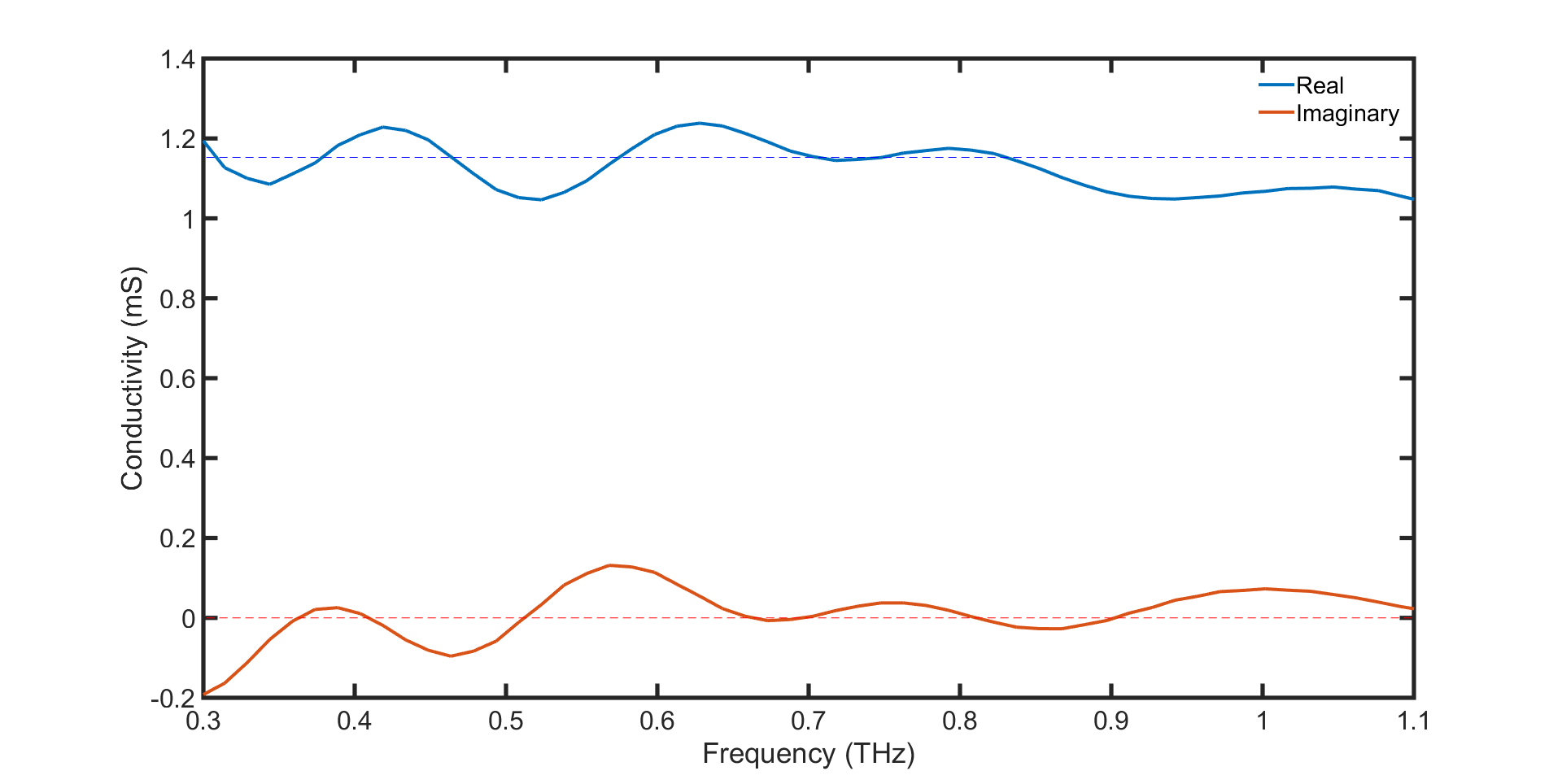


Figure S2– Conductivity spectra measured with THz-TDS operating in reflection-mode. The dotted line correspond to the average value over the spectral range between 0.6-0.9 THz.


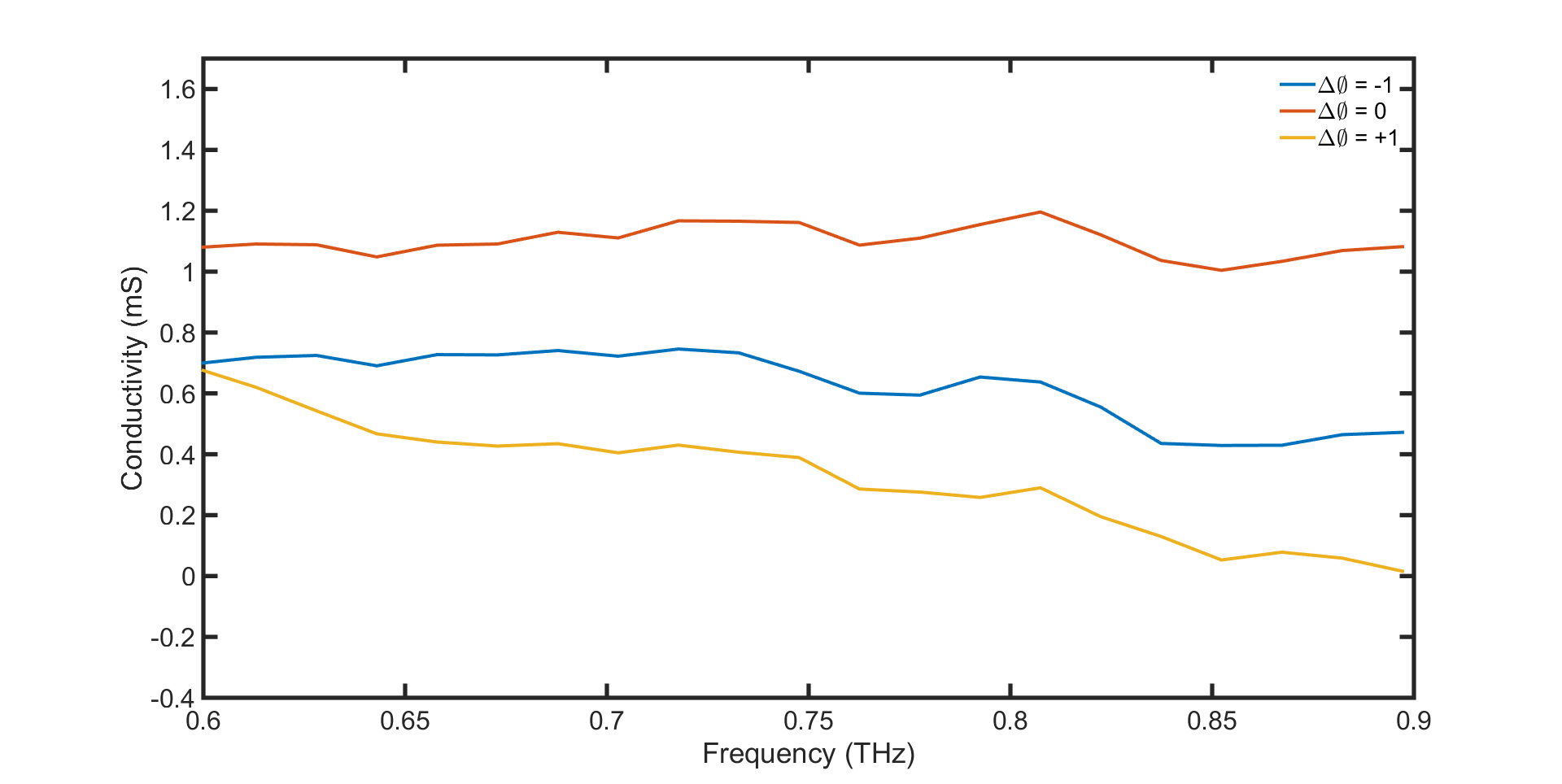


Figure S3 – Conductivity spectrum for graphene on sapphire at three shifted positions of the terahertz pulse relative to the reference mirror as part of the phase correction algorithm where the conductivity spectra with a slope close to zero over 0.6-0.9 THz is selected.


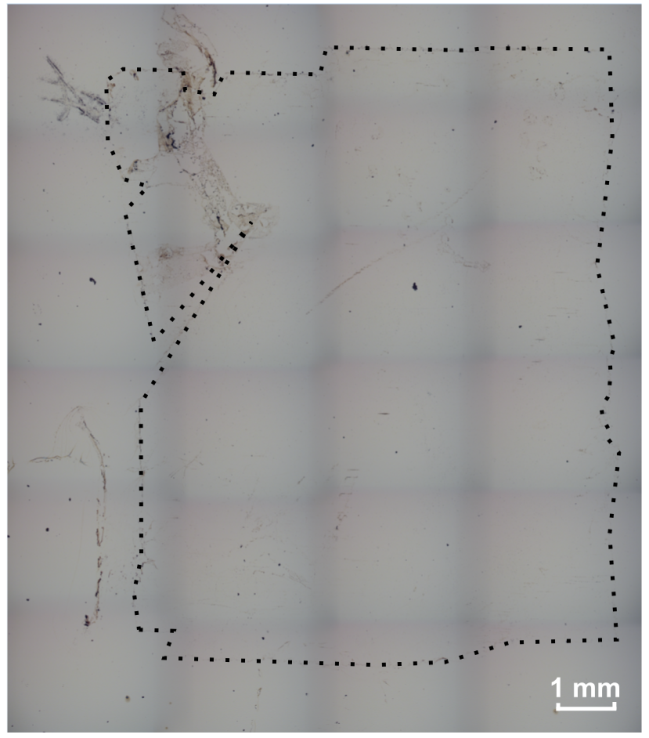


Figure S4 – Stitched microscope photo of the graphene film transferred to an undoped Ge (110) substrate. The optical contrast between graphene covered and uncovered regions is very weak and only distinguishable from the residuals that tend to agglomerate on the edges of the graphene film. The boundary of the graphene covered area is shown by black dots. The square shapes visible in the picture are artefacts from the microscope picture stitching.


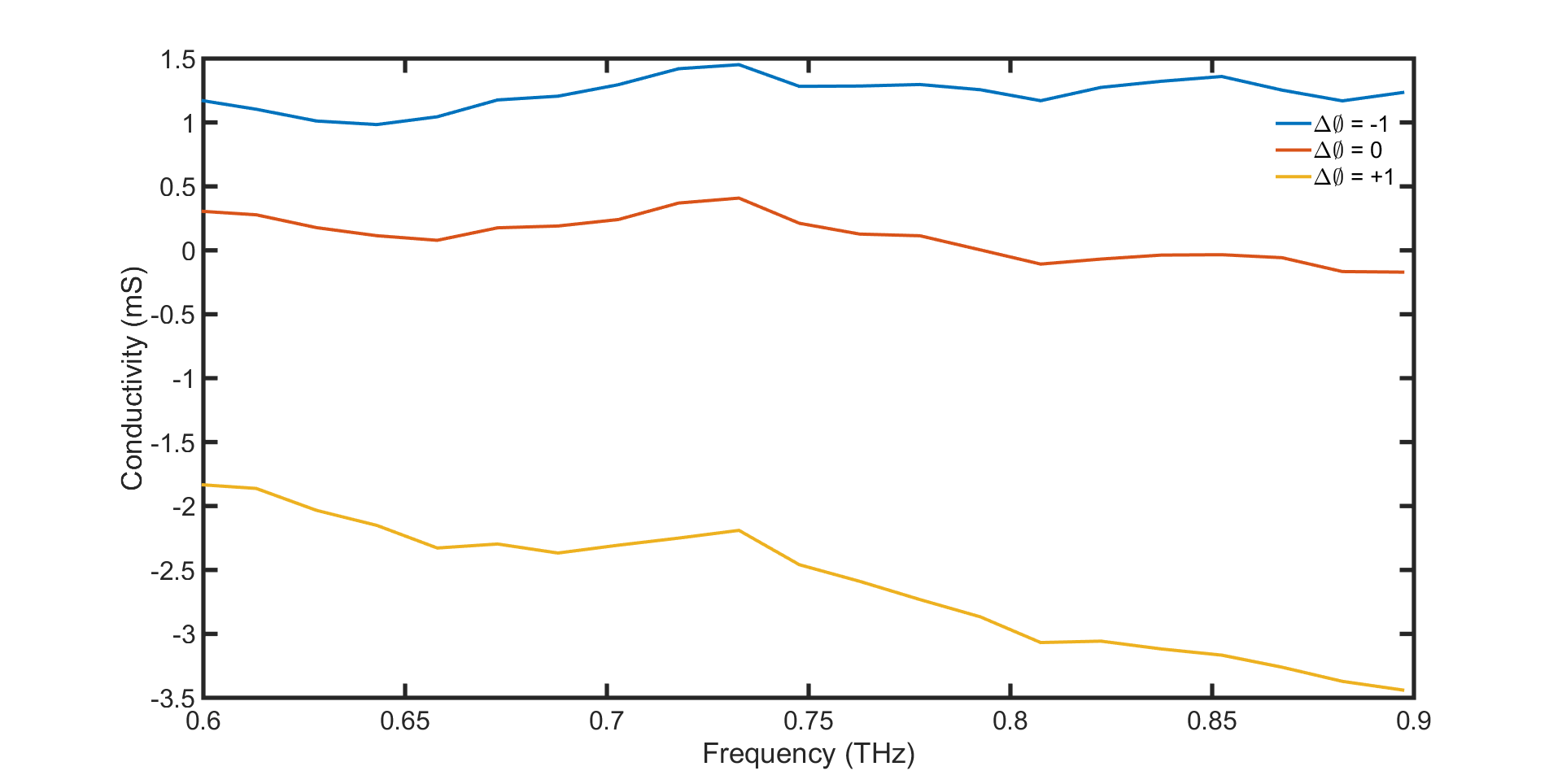


Figure S5 – Conductivity spectrum for graphene on Ge for three shifted positions of the terahertz pulse relative to the reference mirror as part of the phase correction algorithm where the conductivity spectra with a slope close to zero over 0.6-0.9 THz is selected.
